# Being precise with anticoagulation to reduce adverse drug reactions: are we there yet?

**DOI:** 10.1038/s41397-024-00329-y

**Published:** 2024-03-05

**Authors:** Benjamin Cross, Richard M. Turner, J. Eunice Zhang, Munir Pirmohamed

**Affiliations:** 1 Wolfson Centre for Personalised Medicine, Institute of Systems, Molecular and Integrative Biology, The University of Liverpool, 1-5 Brownlow Street, Liverpool, L69 3GL UK; 2 GSK, Stevenage, Hertfordshire SG1 2NY UK

**Keywords:** Personalized medicine, Pharmacogenomics, Genotype, Genetics research, Cardiovascular diseases

## Abstract

Anticoagulants are potent therapeutics widely used in medical and surgical settings, and the amount spent on anticoagulation is rising. Although warfarin remains a widely prescribed oral anticoagulant, prescriptions of direct oral anticoagulants (DOACs) have increased rapidly. Heparin-based parenteral anticoagulants include both unfractionated and low molecular weight heparins (LMWHs). In clinical practice, anticoagulants are generally well tolerated, although interindividual variability in response is apparent. This variability in anticoagulant response can lead to serious incident thrombosis, haemorrhage and off-target adverse reactions such as heparin-induced thrombocytopaenia (HIT). This review seeks to highlight the genetic, environmental and clinical factors associated with variability in anticoagulant response, and review the current evidence base for tailoring the drug, dose, and/or monitoring decisions to identified patient subgroups to improve anticoagulant safety. Areas that would benefit from further research are also identified. Validated variants in *VKORC1*, *CYP2C9* and *CYP4F2* constitute biomarkers for differential warfarin response and genotype-informed warfarin dosing has been shown to reduce adverse clinical events. Polymorphisms in *CES1* appear relevant to dabigatran exposure but the genetic studies focusing on clinical outcomes such as bleeding are sparse. The influence of body weight on LMWH response merits further attention, as does the relationship between anti-Xa levels and clinical outcomes. Ultimately, safe and effective anticoagulation requires both a deeper parsing of factors contributing to variable response, and further prospective studies to determine optimal therapeutic strategies in identified higher risk subgroups.

## Introduction

Anticoagulants are a widely prescribed class of drugs used in both medical and surgical settings. In 2015 alone, 14.6 million prescriptions were dispensed in the community in England, the associated cost was over £222 million which represented an increase of £84 million on the previous year [1, 2].

Oral anticoagulants include indirectly acting coumarin-derived oral anticoagulants, notably warfarin, and direct oral anticoagulants (DOACs)— dabigatran, apixaban, edoxaban and rivaroxaban. In the UK, ~1.25 million patients receive long term oral anticoagulation, and worldwide warfarin remains the most commonly prescribed oral anticoagulant [3]; however warfarin has now been superseded by apixaban as the most widely prescribed oral anticoagulant in primary care in England [4]. Parenteral anticoagulants include indirect acting unfractionated heparin (UFH), low molecular weight heparins (LMWHs), fondaparinux, and the less frequently prescribed direct acting drugs such as bivalirudin and argatroban.

Anticoagulants are primarily indicated in the prophylaxis and treatment of venous thromboembolism (VTE), the prophylaxis of systemic (arterial) thromboembolism in predisposing conditions including atrial fibrillation (AF), following a mechanical heart valve implantation, and in constitutive thrombophilic conditions such as antiphospholipid syndrome. Heuristically, parenteral anticoagulants are preferred for short term inpatient anticoagulation, whilst oral anticoagulants are indicated in longer term outpatient anticoagulation.

Clinically, it is evident that patients can respond differently to the same drug [5]. Precision medicine is a therapeutic paradigm that aspires to tailor drug and/or dose selection to specific patient subgroups to enhance drug efficacy and/or minimise harm. There are three main facets to the *precise* use of therapeutics. First, patients warranting drug intervention need to be identified. For symptomatic conditions patients usually self-present, but asymptomatic conditions require screening. Opportunistic screening for AF for example increases overall AF detection [6], and the use of novel technologies may further facilitate and improve (paroxysmal) AF screening [7]. Second, optimisation of physician conformity to existing therapeutic guidelines is required. For example, ~50% of patients with AF do not receive anticoagulation and of these patients, 25% have no documented reason, suggesting notable anticoagulant underuse [8]. Further initiatives to understand the barriers limiting guideline adherence are necessary.

**Fig. 1:**
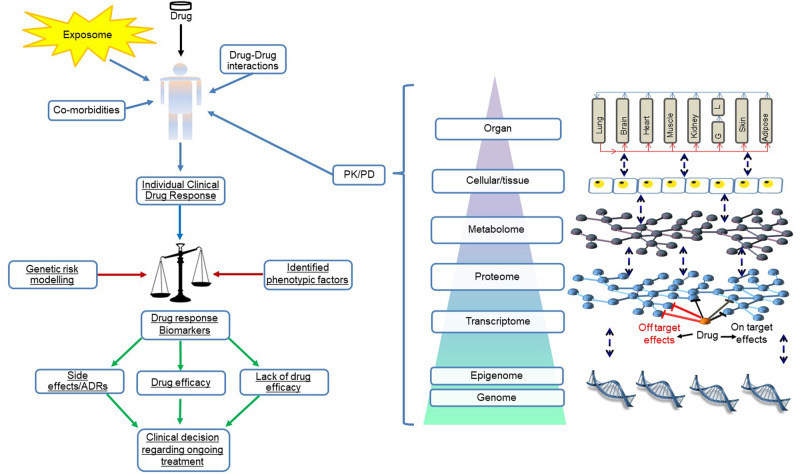
Systems-based approach to Precision Medicine. Interindividual variability in response to a given drug results in efficacy, non-efficacy and adverse drug reactions. The primary sources for this variation are external environmental influences and an individual’s genome and heritable epigenetic traits. The human body consists of a complex dynamic network of interactions within and between its hierarchically organised discrete biological levels (genomic, epigenomic, transcriptomic, proteomic, metabolomic, cellular, tissue, organ, and organ system levels). Consequently, the myriad environmental and heritable traits feed into and shape the human dynamic network; these processes lead to the emergence of non-intuitive system properties including health and disease status and individual drug response. To adequately parse interindividual drug response variability, systematic pan-omics investigations and environmental factor mapping are needed to identify new response biomarkers. Subsequent mechanistic studies will further pathophysiological understanding. Furthermore, it is envisaged that integration of identified factors into algorithms such as polygenic risk scores help define sufficiently distinct patient subgroups, with adequate predictive capacity, which would benefit from distinct treatment strategies. These strategies will include both altered dose and/or drug recommendations with existing therapeutics, and the potential development of novel subgroup-specific therapeutics.

Third, optimisation of drug benefit-risk profiles is required through a deeper understanding of the factors shaping interindividual drug response variability. The human body consists of a complex network of interactions within and between hierarchically organised different biological levels (e.g. genomic, epigenomic, transcriptomic, proteomic, metabolomic, cellular, tissue, organ, and organ system levels), and this dynamic network is shaped by myriad genetic and environment influences. Health, disease and drug response all represent emergent non-intuitive properties arising from this complex network system, and so vary between patients [9]. It is hoped that systematic pan-omics and environmental factor interrogation will identify new drug response biomarkers, and their incorporation into algorithms and multiscale models will parse interindividual drug response variability sufficiently for clinical utility (Fig. 1).

**Fig. 2:**
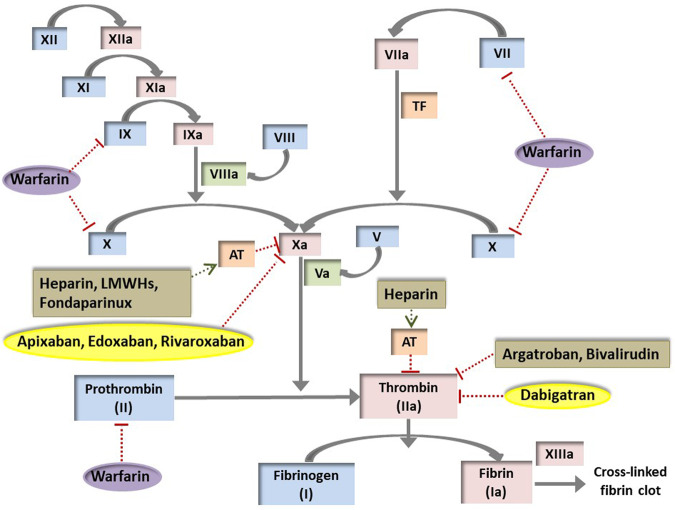
The target sites of oral and parenteral anticoagulants on the clotting system. Vitamin K antagonists (VKAs), notably warfarin, inhibit hepatic vitamin K 2,3 epoxide reductase complex 1 (VKORC1) within the vitamin K cycle. This inhibition decreases active reduced vitamin K, which restricts the post-translational gamma-carboxylation activity of vitamin K-dependent γ-glutamyl carboxylase required by clotting factors II, VII, IX and X to become fully functional. Apixaban, edoxaban and rivaroxaban are direct factor Xa inhibitors, whilst dabigatran inhibits factor IIa (thrombin). A unique pentasaccharide sequence within unfractionated heparin, low molecular weight heparins (LMWHs) and fondaparinux enables high-affinity binding to antithrombin (AT). Heparin chains containing this pentasaccharide sequence and at least 18 saccharide units in length are needed to inactivate factor IIa because bridging to form the ternary heparin/AT/thrombin complex is necessary. Shorter chains containing the pentasaccharide chain can still inactivate Xa and so LMWHs have reduced anti-IIa compared to anti-Xa activity; fondaparinux inhibits Xa but not IIa. Bivalirudin and argatroban are two less frequently used parenterally administered anticoagulants; both are direct thrombin inhibitors. TF tissue factor. Drugs within ovals are orally administered; drugs within rectangles are parenterally administered.

The clotting system balances the opposing needs of free blood flow for tissue viability, with rapid haemostasis following external injury; these competing requirements position the clotting system atop a physiological ‘tightrope’. It is thus highly pertinent to understand interindividual anticoagulant response variability because anticoagulants target and perturb this finely balanced clotting system (Fig. 2) and so their therapeutic window is appreciably narrower than for several other routinely prescribed medications (e.g. lipid lowering therapies, proton pump inhibitors). Individual patient under- and over-anticoagulation risks thrombotic and haemorrhagic complications, respectively, and notably warfarin is third on the list of drugs/drug groups resulting in adverse drug reactions (ADRs) associated with hospitalisation [10]. Furthermore, anticoagulants can also provoke unpredictable ADRs, such as heparin-induced thrombocytopaenia (HIT).

**Fig. 3:**
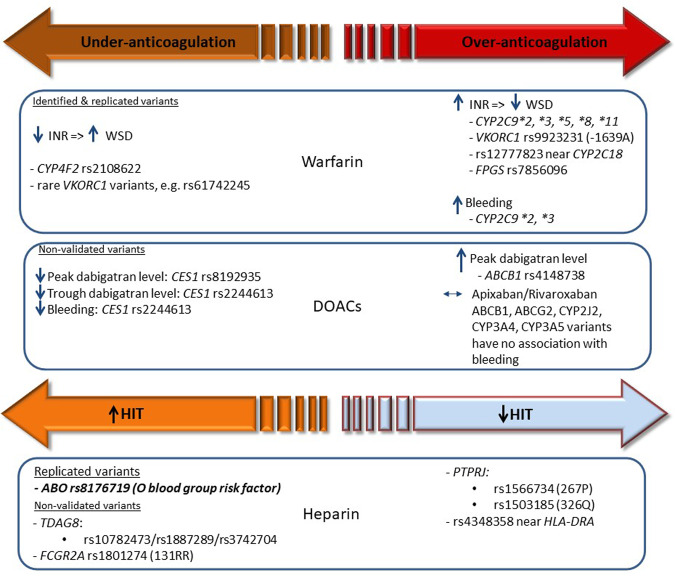
Pharmacogenomic variants associated with differential anticoagulant response. *ABCB1* ATP-binding cassette sub-family B member 1, *CES1* carboxylesterase 1, *CYP2C9* cytochrome P450 2C9, *CYP2C18* cytochrome P450 2C18, *FCGR2A* immunoglobulin G (IgG) receptor IIa gene, *FPGS* folylpolyglutamate synthase, *HLA-DRA* human leucocyte antigen class II, DR alpha, *INR* international normalised ratio, *PTPRJ* protein tyrosine phosphatase receptor type J (CD148), *TDAG8* T-cell death-associated gene 8, *VKORC1* vitamin K 2,3 epoxide reductase complex 1, *WSD* warfarin stable dose.

Therefore, improving the detection of patients that require anticoagulation, increasing observance to existing clinical guidelines, and deepening our understanding of the interindividual variability observed in response to a given anticoagulant are all essential to the long-term realisation of precision anticoagulation. The aims of this review are to discuss the factors associated with interindividual variability in response to warfarin, DOACs, UFH and LMWHs as summarised in Table 1, to consider the current challenges and opportunities for advancing precision anticoagulation, and to highlight areas of unmet research need. Figure 3 highlights the main pharmacogenomic variants associated with differential response to anticoagulants.

**Table 1:** Drug-specific factors that have been associated with interindividual variability in response to anticoagulation therapy.

Drug	Outcome	Factor	Effect	References
Oral anticoagulants				
Warfarin	Stable anticoagulation	Age	~8–10% dose reduction per decade	[16, 30]
		Amiodarone	30% dose reduction	[18, 46]
		Smoking	Increased warfarin dose requirement	[22]
		CYP2C9*2, *3, or *2/*3	Decreased warfarin dose requirement; explain 10–15% dose variability	[16, 23, 28–31]
		CYP2C9*5 rs28371686	Decreased warfarin dose requirement	[23]
		CYP2C9*8 rs7900194	Decreased warfarin dose requirement	[23]
		CYP2C9*11 rs28371685	Decreased warfarin dose requirement	[23]
		rs12777823, near 5′ end of *CYP2C18*	Decreased warfarin dose requirement	[34]
		*FPGS* rs7856096	Decreased warfarin dose requirement	[45]
		*VKORC1* rs9923231	Increased warfarin dose requirement; explain 20–35% dose variability	[28–31]
		*CYP4F2* rs2108622	Increased warfarin dose requirement; explain 1–7% dose variability	[35, 44, 231]
	Time to therapeutic INR	*VKORC1* haplotypes	Decreased time	[40]
	INR ≥ 4	CYP2C9*2, *3, or *2/*3	Increased risk	[27, 31, 232]
		*VKORC1* rs9923231	Increased risk	[31, 40]
	Bleeding	CYP2C9*2, *3, or *2/*3	2–3 fold increased risk. CYP2C9*3/*3 versus CYP2C9*1/*1, HR 4.87 (95% CI: 1.38–17.14)	[26, 27]
Dabigatran	Bleeding	*CES1* rs2244613	Reduced risk; RR 0.67 (95% CI: 0.55–0.82) per minor allele	[126]
	Major bleeding	P-gp inhibitor (amiodarone 200 mg/day)	25-fold increase in dabigatran trough concentration; major bleeding led to haemorrhagic shock and subsequent death (case report)	[73, 233]
		P-gp inhibitor (dronedarone)	Acute left temporoparietal SDH, with SAH and trace IVH (case report)	[72]
		Concomitant antiplatelet therapy	Dabigatran 110 mg: HR 2.14 (95% CI: 1.75–2.61) Dabigatran 150 mg: HR 2.05 (95% CI: 1.66–2.54)	[83]
		Dual antiplatelets therapy	HR 2.31 (95% CI: 1.79–2.98)	[83]
		Dabigatran 150 mg twice daily (higher rates of bleeding compared to 110 mg twice daily)	Rates of major haemorrhage were 3.74% and 2.99%/year for dabigatran 150 and 110 mg (HR 1.26 (95% CI: 1.04–1.53)	[234]
	Gastrointestinal bleeding	65–74 years of age; ≥75 years of age	HR 2.72 (95% CI: 1.59–4.65); HR 4.52 (95% CI: 2.68–7.64)	[74]
		Male	HR 0.78 (95% CI: 0.64–0.95)	[74]
		Congestive Heart Failure	HR 1.25 (95% CI: 1.01–1.56)	[74]
		Renal impairment	HR 1.67 (95% CI: 1.24–2.25)	[74]
		Alcohol abuse	HR 2.57 (95% CI: 1.52–4.35)	[74]
		Previous *Helicobacter pylori* infection	HR 4.75 (95% CI: 1.93–11.68)	[74]
		Antiplatelet agent (including prescription aspirin)	HR 1.49 (95% CI: 1.19–1.88)	[74]
		Digoxin	HR 1.33 (95% CI: 1.05–1.68)	[74]
	Stroke	Obesity	Increased GFR and reduced trough plasma level	[92, 93]
	PE	Obesity	Increased risk	[235]
	Trough plasma level	Weight > 100 kg	20% decrease in the trough levels of dabigatran	[87]
		*CES1* rs2244613	15% decrease per minor allele; *P* = 1.2 × 10^−8^ (95% CI:10–19)	[126]
	Peak plasma level	*CES1* rs8192935	12% decrease per minor allele; *P* = 3.2 × 10^−8^ (95% CI: 8–16)	[126]
		*ABCB1* rs4148738	12% increase per minor allele; *P* = 8.2 × 10^−8^ (95% CI: 8–17)	[126]
	Bioavailability	P-gp inhibitors	10–20% increase in dabigatran bioavailability	[71]
	Exposure	CrCl 15–30 mL/min	6.3 fold increase	[118]
		CrCl 30–50 mL/min	3.2 fold increase	[118]
Rivaroxaban	Clinically relevant bleeding	Aspirin	HR 1.81 (95% CI:1.36–2.41)	[84]
		NSAIDs	HR 1.90 (95% CI:1.45–2.49)	[84]
	Major bleeding	Aspirin	HR 1.50 (95% CI:0.63–3.61)	[84]
		NSAIDs	HR 2.56 (95% CI:1.21–5.39)	[84]
	Exposure	CrCl <30 mL/min	65% increase	[119]
		CrCl 30–49 mL/min	52% increase	[119]
		CrCl 50–79 mL/min	44% increase	[119]
Apixaban	Major bleeding	Age	HR 1.36 (95% CI:1.23–1.51) per decade increase	[85]
	All-cause mortality	BMI 25–29; BMI > 30 (vs BMI < 25)	HR 0.65 (95%CI: 0.57–0.75); HR 0.61 (95% CI: 0.52–0.71)	[236]
	Composite event rates (stroke/systemic embolism, myocardial infarction and/or all-cause mortality)	BMI 25–29; BMI > 30 (vs BMI < 25)	HR 0.72 (95%CI: 0.64–0.81); HR 0.67 (95% CI: 0.58–0.76)	[236]
	Exposure	CrCl 15–29 mL/min	44% increase	[121]
		CrCl 30–50 mL/min	29% increase	[121]
		CrCl 51–80 mL/min	16% increase	[121]
Edoxaban	Major bleeding	Antiplatelet therapy	Increased incidence of major bleeding compared to patients not receiving antiplatelet therapy; 3.55% per year versus 2.04% per year	[86]
		Number of dose adjustment factors (CrCl 15–50 mL/min, weight <60 kg, concomitant medication with potent P‐glycoprotein inhibitors) in patients on 30 mg/day	Increased major bleeding in those with 3 vs 0–1 dose adjustment factors. Suggested increased major bleeding with 2 vs 0–1 factors.	[237]
	Exposure	CrCl <30 mL/min	72% increase	[120]
Parenteral anticoagulants				
Unfractionated heparin	Markedly low initial aPTT (<1.25x control) in STEMI	Increased weight	RR 1.23 (95% CI 1.15–1.31) per 10 kg increase	[156]
		Younger age	RR 1.42 (95% CI 1.31–1.54) per decade decrease	[156]
		Female	RR 1.55 (95% CI 1.24–1.95)	[156]
	Markedly high initial aPTT (≥2.75x control) in STEMI	Increased age	RR 1.14 (95% CI 1.06–1.23) per decade increase	[156]
		Female	RR 1.46 (95% CI 1.21–1.78)	[156]
		Lower weight	RR 1.19 (95% CI 1.11–1.27)	[156]
		Renal dysfunction	RR 1.08 (95% CI 1.02–1.13) per 0.2 mg/dL increase in serum creatinine	[156]
	180 day VTE recurrence after incident VTE	Attainment of aPTT ≥58 s within 24 ± 4 h of starting UFH^i^	HR 0.57 (95% CI 0.34–0.97)	[155]
		Proportion of time with aPTT ≥40 s per 10% increase	HR 0.90 (95% CI 0.83–0.97)	[155]
	MI recurrence following STEMI	Markedly low aPTTs (<1.25x control) taken 4–6 h after starting UFH therapy^i^	OR 3.0 (95% CI 1.28–7.04)	[156]
	Heparin resistance	Antithrombin deficiency, Platelet count >300,000/microL, Recent heparin therapy, Increased levels of heparin-binding proteins, increased heparin clearance, high levels of factor VIII and fibrinogen, concomitant use of aprotinin	Increased risk of heparin resistance	[144, 160, 162, 163]
	Bleeding	Markedly high initial aPTT (≥2.75x control) in STEMI^i^	OR 1.72 (95% CI 0.98–3.00)	[156]
	Heparin-induced thrombocytopaenia	UFH compared to LMWH in surgical patients	OR 0.10 (95% CI 0.01–0.2)	[171]
		Therapeutic UFH	IV therapeutic UFH: 0.76% develop HIT SC prophylactic UFH: <0.1% develop HIT	[173]
		Female	OR 2.37 (95 CI 1.37–4.09)	[174]
		*TDAG8* rs10782473	OR 18.52 (95% CI 6.33–54.23)	[178]
		*HLA-DRA* rs4348358	OR 0.25 (95% CI 0.15–0.44)	[178]
		*PTPRJ* rs1566734 (Q267P)	OR 0.36 (95% CI 0.20–0.67)	[176]
		*PTPRJ* rs1503185 (R326Q)	OR 0.37 (95% CI 0.20–0.68)	[176]
	Thrombosis in HIT	*FCGR2A* rs1801274 (H131R)	OR 5.9 (95% CI 1.7–20)	[177]
	Hyperkalaemia	Longer hospitalisation duration	Increased risk (*p* < 0.05)	[185]
		Baseline serum potassium	OR 43.1 (95% CI 1.40–45.76)	[181, 185]
		Diabetes mellitus	Increased risk (*p* < 0.001)	[185]
		Metabolic acidosis	Increased risk (*p* < 0.005)	[185]
		Renal dysfunction	Increased risk	[182, 238]
	Osteoporosis	Long term UFH therapy in pregnant patients	Bone mineral density reduction (≤30% patients) Osteoporotic fractures (2.2–5% patients)	[239, 240]
Low molecular weight heparins	Low anti-Xa activity	Weight	A negative correlation between body weight and anti-Xa activity (Spearman’s rho = −0.428)	[191]
		Peripheral oedema	Anti-Xa AUC_0–12 h_ if oedematous: 0.63 IU/L Anti-Xa AUC_0–12 h_ if non-oedematous: 1.57 IU/L (*p* = 0.001)	[205]
		Multiple organ dysfunction	OR 1.56 (95% CI 1.15–2.12)	[206]
		COVID-19	Decreased recovery of anti-Xa of the COVID19 patients assessed, *p* < 0.05 (*t* test), with the maximum recovery being 82% and the minimum recovery being 58% when compared with a calculated expected recovery.	[241]
	High anti-Xa activity	Weight ≤45 kg vs 51–55 kg	OR 5.52 (95% CI 0.91–33.57)	[191]
		Renal dysfunction	Enoxpararin Thromboprophylaxis: significant accumulation Therapeutic: significant accumulation	[193]
			Nadroparin Thromboprophylaxis: no accumulation Therapeutic: significant accumulation	[223, 224]
			Dalteparin Thromboprophylaxis: no accumulation Therapeutic: no accumulation	[193, 227]
			Tinzaparin Thromboprophylaxis: no accumulation Therapeutic: no accumulation	[225, 226]
		Body weight ≤45 kg vs 51–55 kg	OR 5.52 (95% CI 0.91–33.57)	[191]
	DVT in high risk surgical and trauma patients	Low trough anti-Xa (≤0.1 IU/mL) activity^iii^	Increased DVT risk (*p* = 0.026)	[207]
	Bleeding	Therapeutic enoxaparin with CrCl ≤ 30 mL	OR 3.88 (95% CI 1.78–8.45)	[230]
		anti-Xa activity >0.9 IU/mL^ii^	OR 1.6 (95% CI 1.0–2.5) per unit of anti-Xa	[201]

i = the associations between aPTT on heparin and thrombotic or haemorrhagic endpoints are inconsistent and have not been observed in other studies; ii = the associations between anti-Xa on low molecular weight heparin and thrombotic or haemorrhagic endpoints are inconsistent and have not been observed in other studies.

*ABCB1* ATP-binding cassette sub-family B member 1, *aPTT* activated partial thromboplastin time, *AUC* area under the curve, *BMI* body mass index, *CES1* carboxylesterase 1, *CI* confidence interval, *CrCl* creatinine clearance, *CYP* cytochrome P450, *DVT* Deep vein thrombosis, *FCGR2A* Fc fragment of IgG, low affinity IIa, receptor, *FPGS* folylpolyglutamate synthase, GFR glomerular filtration rate, HIT heparin-induced thrombocytopaenia, *HLA-DRA* human leucocyte antigen class II, DR alpha, *HR* hazard ratio, *INR* international normalised ratio, *IU* International units, *IVH* intraventricular haemorrhage, *IV* Intravenous, *LMWH* low molecular weight heparin, *MI* myocardial infarction *OR* odds ratio, *PE* pulmonary embolism, *P-gp* permeability glycoprotein, *PTPRJ* protein tyrosine phosphatase receptor type J (CD148), *RR* relative risk, *SAH* subarachnoid haemorrhage, *SC* subcutaneous, *SDH* subdural haematoma; *STEMI* ST segment elevation myocardial infarction; *TDAG8* T-cell death-associated gene 8, *UFH* unfractionated heparin, *VKORC1* vitamin K 2,3 epoxide reductase complex 1, *VTE* venous thromboembolism.

## Oral anticoagulants

### Warfarin

The coumarin-derived racemic mixture, warfarin, at peak usage was estimated to be taken by at least 1% of the whole UK population, and by 8% of those aged over 80 years [11]. However, warfarin usage has decreased in many European countries and in the US, with a concomitant increase in the use of DOACs. Nevertheless, warfarin is still widely used especially in some of the lower-middle income countries, where affordability is a major issue.

Warfarin inhibits hepatic vitamin K 2,3 epoxide reductase complex 1 (VKORC1). VKORC1 is the rate-limiting enzyme in the warfarin sensitive vitamin K-dependent gamma carboxylation system, and inhibition of VKORC1 reduces the production of functional clotting factors II, VII, IX and X, proteins C, S and Z, and leads to anticoagulation (Fig. 2). Warfarin has a narrow therapeutic window and large inter-individual variability with up to 20-fold difference in stable dose requirements between individuals. Therefore, warfarin treatment is closely monitored via the international normalised ratio (INR); for most indications, the recommended therapeutic INR range is 2.0–3.0. An overview of warfarin pharmacokinetics is provided in Table 2.

**Table 2:** Pharmacological characteristics of warfarin and DOACs.

Drug	Warfarin	Dabigatran	Rivaroxaban	Apixaban	Edoxaban
Commercial name (licenser)	COUMADIN^®^ (Bristol-Myers-Squibb)	PRADAXA^®^ (Boehringer Ingelheim Pharma GmbH & Co. KG)	XARELTO^®^ (Bayer Pharma AG)	ELIQUIS^®^ (Bristol-Myers-Squibb and Pfizer)	LIXIANA^®^ or SAVAYSA^®^ (Daiichi Sankyo)
Empirical Formula	C_19_H_16_O_4_	C_35_H_45_N_7_O_8_S (mesylate salt)	C_19_H_18_ClN_3_O_5_S	C_25_H_25_N_5_O_4_	C_24_H_30_ClN_7_O_4_S•C_7_H_8_O_3_S•H_2_O
Molecular weight (g/mol)	308.33	723.86	435.89	459.5	738.27
Prodrug	No	Yes	No	No	No
Mechanism of action	Reduced synthesis of prothrombin and other vitamin K-dependent factors	Direct inhibition of thrombin	Direct inhibition of Factor Xa	Direct inhibition of Factor Xa	Direct inhibition of Factor Xa
Indication	• Prevention and treatment of thromboembolic complications associated with AF and/or cardiac valve replacement. • Prevention and treatment of DVT and PE • Reduce risk of recurrent DVT and PE • Reduce risk of death, recurrent MI, and thromboembolic events after MI.	• Prevention of thromboembolic complications in NVAF • Treatment of DVT and PE • Prevention of DVT and PE • Reduce risk of recurrent DVT and PE	• Prevention of thromboembolic complications in NVAF • Treatment of DVT and PE • Prevention of DVT and PE • Reduce risk of recurrent DVT and PE	• Prevention of thromboembolic complications in NVAF • Treatment of DVT and PE • Prevention of DVT and PE • Reduce risk of recurrent DVT and PE	• Prevention of thromboembolic complications in NVAF • Treatment of DVT and PE
Oral dosing (frequency)	Variable dose adjusted based on INR (Once per day)	Fixed dose (Once or twice per day, dependent on indication)	Fixed dose (Once or twice per day, dependent on indication)	Fixed dose (Twice per day)	Fixed dose (Once per day)
Dosage strengths available	0.5 mg, 1 mg, 3 mg, 5 mg	75 mg, 110 mg, 150 mg	10 mg, 15 mg, 20 mg	2.5 mg, 5 mg	15 mg, 30 mg, 60 mg
Dose-dependent pharmacokinetics (linear PK)	Yes	Yes	Yes	Yes	Yes
Bioavailability (%)	98	3–7	≥80 (Optimal drug absorption is achieved when taken with food)	50	62
Peak onset (h)	72–120	0.5–2 (aged 18–45 years) 2–4 (aged ≥65 years)	2–4	1–3	1–2
Plasma half-life (h)	20–60	7–9 (single dose) 14–17 (multiple doses, aged 18–45 years) 12–14 (multiple doses, aged ≥65 years)	7–11 (aged 20–45 years) 11–13 (aged ≥60 years)	8–15	10–14
Protein binding (%)	99%	35%	92–95%	87%	40–59%
Volume of distribution (L)	Yes	50–70	50	21–23	>300
P-gp substrate	Yes	Yes	Yes	Yes	Yes
Metabolism	Oxidation (mainly via CYP2C9 with minor contributions from CYP1A2, 2C8, 2C18, 2C19, 3A4)	Conjugation	Oxidation via CYP3A4/5, 2J2 (major pathway) and hydrolysis	Oxidation (mainly via CYP3A4/5 with minor contributions from CYP1A2, 2J2, 2C8, 2C9, 2C19) and conjugation	Hydrolysis via CES1 (major pathway) and oxidation via CYP3A4
Renal excretion of unchanged drug (%)	0	77^a^ 4^b^	36	27	50
Food and alcohol interaction	Yes	No	No	No	No
Dose monitoring	PT/INR	Not required Useful tests • aPTT: approximate measurement of drug effect • dTT: quantitative plasma drug level • ECT: quantitative plasma drug level	Not required Useful tests • PT: approximate measurement of drug effect • Chromogenic anti-Factor-Xa assay: quantitative plasma drug level	Not required Useful tests • PT: approximate measurement of drug effect • Chromogenic anti-Factor-Xa assay: quantitative plasma drug level	Not required Useful tests • PT: approximate measurement of drug effect • Chromogenic anti-Factor-Xa assay: quantitative plasma drug level
Antidote	• Rapid reversal with fresh-frozen plasma or PCC • Slow reversal with vitamin K	• Rapid reversal with idarucizamab • Aripazine (ongoing phase II trial)	• Adexanet-α (ongoing phase IV trial) • Aripazine (ongoing phase II trial)	• Adexanet-α (ongoing phase IV trial) • Aripazine (ongoing phase II trial)	• Adexanet-α (ongoing phase IV trial) • Aripazine (ongoing phase II trial)
References	[242]	75, 79, 115, 136, 138, 139, 243–247]	[82, 117, 142, 248–257]	[80, 116, 140, 141, 256–264]	76, 81, 140, 141, 256, 257, 265–269]

*AF* atrial fibrillation, *aPTT* activated partial thromboplastin time, *CES1* carboxylesterase 1, *CYP* cytochrome P450, *DVT* deep vein thrombosis, *INR* international normalised ratio, *MI* myocardial infarction, *NVAF* nonvalvular atrial fibrillation, *PCC* prothrombin complex concentrate, *PT* prothrombin time.

^a^After intravenous administration of dabigatran.

^b^After oral administration of dabigatran.

Patients with AF on warfarin are unsettlingly outside the therapeutic INR range 30–50% of the time [12, 13]. Importantly, bleeding is the most common warfarin ADR occurring in up to 41% of treated patients, with major bleeding frequencies as high as 10–16% [14, 15]. The risk of adverse events is highest during the initial dose-titration period within the first few weeks to months of warfarin therapy, and so strategies to individualise the initial warfarin doses have been sought.

#### Clinical and environmental factors affecting warfarin response

Numerous clinical and environmental factors influence warfarin dose requirements and response, including age, ethnicity, weight, height, medications, diet, illness, smoking and crucially adherence.

Increasing patient age has consistently been associated with higher warfarin sensitivity, which may be caused by the significant negative correlation between age and warfarin clearance, and by the fall in total hepatic VKORC1content due to age-related decreases in hepatic mass requirements [16].

Concomitant medications can affect warfarin pharmacokinetics by reducing its intestinal absorption, altering its clearance, or by competing for protein binding. Drugs can also influence the pharmacodynamics of warfarin by mechanisms such as inhibition of the synthesis of vitamin K-dependent coagulation factors or increasing the clearance of these factors. A list of major medications that interact with warfarin has been reviewed [17]. Importantly, patients on amiodarone require 20–30% lower doses of warfarin for stable anticoagulation [18].

Dietary factors can affect warfarin dose requirements, such as alcohol consumption or vitamin K intake. Alcohol may perturb warfarin metabolism and high dietary intake of vitamin K (found in green vegetables) may conceivably offset warfarin activity. However, there is conflicting evidence on the association between warfarin maintenance doses and vitamin K intake [19, 20].

Several illnesses such as liver disease, malnutrition, decompensated heart failure, hypermetabolic states (e.g. febrile illnesses, hyperthyroidism) are recognised to affect warfarin dose requirements [18, 21].

Cigarette smoking can induce CYP1A2 activity, the major enzyme responsible for *R*-warfarin metabolism. With increased smoking, *R*-warfarin metabolism increases, increasing dose requirements. Therefore, a change in smoking habit may affect warfarin coagulation response and consequently patients should be carefully monitored and warfarin doses reduced accordingly following cessation [22].

#### Genetic factors affecting warfarin dose requirements

##### CYP2C9

CYP2C9 metabolises the S-warfarin enantiomer, which is 3-5x more potent than R-warfarin. Over 30 *CYP2C9* variants are recognised, although CYP2C9*2, *3, *5, *6, *8 and *11 represent the main *CYP2C9* non-synonymous reduction-of-function (ROF) single nucleotide polymorphisms (SNPs). These SNPs all attenuate S-warfarin metabolism, although CYP2C9*6 is an exonic single nucleotide deletion, which shifts the reading frame and leads to complete loss of function [23]. CYP2C9*2 and *3 are the most common Caucasian variants with minor allele frequencies of 0.13 and 0.07, respectively. In Asian populations, CYP2C9*2 is very rare and CYP2C9*3 has a low frequency (~0.04); in African populations CYP2C9*2 and *3 are both rare or absent. CYP2C9*2 and *3 reduce S-warfarin metabolism by ~30–40% and ~80–90% respectively [24], and are associated with both decreased WSD requirements [25] and an increased risk of bleeding [26, 27]. The largest bleeding risk is apparent in patients homozygous for CYP2C9*3, with a hazard ratio for bleeding relative to CYP2C9*1/*1 patients of 4.87 (95% confidence interval 1.38, 17.14) [26]. Using multiple linear regression models, several observational studies have shown that *CYP2C9* polymorphisms account for ~10–15% of the variance in warfarin maintenance dosage [16, 28–31].

The variants, CYP2C9*5, *6, *8 and *11, are present mainly in African populations. With the exception of CYP2C9*6 for which there is presently insufficient evidence, CYP2C9*5, *8 and *11 are all associated with reduced warfarin dose requirements [32, 33]. Interestingly, a genome-wide association study (GWAS) identified an intergenic SNP, rs12777823, located near the 5’ end of *CYP2C18* within the *CYP2C* gene cluster, that was associated with lower warfarin dose requirements in African-American patients [34]. Incorporation of rs12777823 improved the proportion of warfarin dose variability in these patients by an absolute of 5% [34].

##### VKORC1

The common *VKORC1* SNP, rs9923231(c.-1639G>A), has consistently been associated with reduced warfarin dose requirements [34–36]. -1639A perturbs a transcription factor binding site in the *VKORC1* promoter region and reduces gene expression [37]. In African-American, Asian and Caucasian populations, the allele frequency of -1639A is ~0.13, ~0.92 and ~0.40 respectively, indicating reversal of the minor allele within Asian populations. rs9923231 accounts for 20–25% of WSD variation in Asian and Caucasian populations, but only ~6% in African-Americans [38]. This is potentially attributable to both its lower frequency and/or the influence of additional factors in Africa-American patients. rs9923231 has been associated with an increased risk of bleeding in some studies [31, 39, 40], but not others [41, 42]. Interestingly, several rare *VKORC1* mutations (e.g. rs61742245, D36Y) have been identified in patients resistant to warfarin that require high warfarin doses to achieve therapeutic anticoagulation [43].

##### CYP4F2

A non-synonymous variant (rs2108622) in the vitamin K oxidase gene, *CYP4F2*, associated with increased warfarin dose requirements has been confirmed in genome-wide studies [35, 36]. CYP4F2 metabolises reduced (active) vitamin K, removing it from the vitamin K cycle. rs2108622 accounts for 1–7% of dose variance [35, 44].

### Other genetic factors

Interestingly, a population-specific regulatory variant (rs7856096) located in the folate homoeostasis gene folylpolyglutamate synthase (FPGS) was identified through exome-sequencing of African-American patients with extreme warfarin dose requirements, and was associated with lower warfarin dose requirements [45].

There are other genes that might potentially influence warfarin response but these have not been consistently identified in different studies, and have not been identified in genome-wide association studies. That does not mean that they are not important, but it is possible that their effect size is much lower than the 3 main genes so far identified to affect warfarin response. Much larger studies would be needed to consistently detect their effect.

#### Warfarin genetic testing

Together, *CYP2C9* and *VKORC1* SNPs and clinical variables account for nearly 60% of warfarin dose variance [31, 46]. Despite results from many multiple regression analyses demonstrating that genetic information from *CYP2C9* and *VKORC1* provides good predictive power with regards to warfarin dosage, there is currently no recommendation for genetic screening of patients starting warfarin therapy in guidelines from the cardiology and thoracic societies, although CPIC has provided detailed advice on dose changes in people with different CYP2C9 and VKORC1 variants [47]. A handful of randomised controlled trials have attempted to evaluate whether applying pharmacogenomic dosing algorithms to clinical practice translates into better clinical outcomes, such as more rapid attainment of therapeutic INR or a reduction in percentage of out-of-range INR. ENGAGE-AF-TIMI 48 [48] trial demonstrated patients with AF receiving clinical based warfarin dosing who were deemed sensitive and highly sensitive responders to warfarin on genetic testing (incorporating CYP2C9 (*2 and *3 alleles; rs1799853 and rs1057910) and VKORC1 (-1639G->A; rs9923231)) were more likely to bleed and have raised INRs. This result was consistent even after adjustment for clinical co-variates [49]. Sub analysis of Hokusai VTE trial echoed these findings with pooled sensitive responders spending more time with higher INRs and increased bleeding events [50]. The EU-PACT [51] trial showed that pharmacogenomic-guided dosing was superior to fixed dosing regimen but the COAG [52] trial did not. Reasons for this divergence in outcome were largely due to ethnicity of patients (27% African-American in COAG versus almost 100% Caucasians in EU-PACT), and the availability of genotype data prior to warfarin initiation. The results of the EU-PACT trial were confirmed by an implementation study which also utilised point of care warfarin genetic testing and showed an improvement in the time in therapeutic range compared to standard of care [53]. Furthermore, the GIFT [54] trial supported the findings of EU-PACT again in a predominantly Caucasian based more elderly cohort demonstrating reduction in bleeding endpoints, less time with INR > 4 and non-inferior protection against VTE.

The potential utility of warfarin genotype-guided dosing has also been shown in two real world evaluations. In Finland, an evaluation of warfarin treated patients from a biobank demonstrated that sensitive and highly sensitive responders spent a longer time with supratherapeutic INRs but there was no significant increase in bleeding risk, although there were few bleeding events in the study [55]. A retrospective cohort study in the US showed that pharmacist-guided warfarin service which utilised pharmacogenetic-guided dosing was able to reduce warfarin-related hospitalisations [56].

Most of the studies on warfarin pharmacogenetics have been conducted in European ancestry patients. However, our systematic review showed that there has been significant activity in developing dosing algorithms for individuals of Asian ancestry, in addition to European ancestry patients [57]. Indeed, up till May 2020, 433 dosing algorithms have been described in the literature, but the majority have not been evaluated for clinical utility. The covariates included in these algorithms have been age (included in 401 algorithms), concomitant medications (270 algorithms), weight (229 algorithms), CYP2C9 variants (329 algorithms), VKORC1 variants (319 algorithms) and CYP4F2 variants (92 algorithms).

There has been much less work on developing algorithms in individuals of African ancestry than in other populations [57]. A systematic review has shown that variants which are more prevalent in Black Africans have functional effects which are equivalent to those seen in White individuals [58], yet these have not been routinely utilised in dosing algorithms, nor tested prospectively in randomised trials. In the COAG trial [52], Black patients were shown to have worse anticoagulation control when randomised to the genotyping arm compared to the use of the clinical algorithm—this is likely to have been due to the lack of African-specific variants in the dosing algorithm. Indeed a recent study has shown that pharmacogenetic dosing algorithms that did not incorporate CYP2C9*5 overestimated the warfarin dose by 30% [59]. Given the widespread usage of warfarin in Black patients, particularly in Sub-Saharan Africa where DOACs are still unaffordable, it is important further studies are undertaken to improve the quality of anticoagulation with warfarin in this population.

### Direct oral anticoagulants

Over the past decade, DOACs have emerged as oral anticoagulant alternatives to warfarin. DOACs reversibly target the active sites of circulating and clot-bound thrombin (dabigatran) or clotting factor Xa (rivaroxaban, apixaban and edoxaban) (Fig. 2). Compared to warfarin, DOACs have a rapid onset of action, a wider therapeutic window, and fewer food and drug interactions. Currently, DOACs are prescribed at fixed doses without laboratory monitoring. However, clinical and genetic factors have been shown to affect DOAC efficacy and safety and dose adjustments may be required in high-risk patients. An overview of DOAC pharmacokinetics is provided in Table 2.

#### Efficacy and safety of DOACs compared with standard treatment

In non-valvular AF, a meta-analysis of the four main trials investigating dabigatran, rivaroxaban, apixaban, and edoxaban revealed that the rate of stroke and systemic embolism, all-cause mortality and intracranial haemorrhage were all significantly reduced by 19%, 10%, and 52%, respectively, compared to patients on warfarin [60]. However, with the exception of apixaban; rivaroxaban, higher doses of dabigatran and edoxaban were associated with 25% increased risk of gastrointestinal bleeding [60, 61].

Meta-analysis of trials that investigated the efficacy and safety of dabigatran, rivaroxaban, apixaban, and edoxaban in patients with acute VTE, demonstrated DOACs were non-inferior to conventional therapy and associated with a reduced risk of bleeding [62]. Pooled analysis of trials conducted with dabigatran, rivaroxaban, apixaban and edoxaban revealed that DOACs are effective for post-operative thromboprophylaxis in patients after a total hip or knee replacement, but their clinical benefits over LMWHs are marginal and DOACs are generally associated with higher bleeding tendency [63, 64].

Finally, in a phase II dose validation study (RE-ALIGN), the efficacy and safety of dabigatran was compared to warfarin in patients with mechanical heart valves [65]. The study was however terminated prematurely due to increased incidence of thromboembolic and bleeding events in the dabigatran-treated patients and the thromboembolic effects were seen in patients with both high and low trough levels [66]. Therefore, whilst DOACs are indicated in the management of non-valvular AF and VTE, warfarin currently remains the drug of choice for patients with mechanical heart valves. Three small scale proof of concept trials with rivaroxaban in patients with mechanical heart valves demonstrate future investigation of DOACs in mechanical heart valves may warrant investigation [67–69].

#### Factors affecting efficacy and safety of DOACs

##### Food and drug interactions

Unlike warfarin, DOACs are not known to be affected by food and have fewer drug-drug interactions. A comprehensive list of drug interactions with DOACs has been reviewed by Heidbuchel et al. [70].

##### P-gp inhibitors, inducers and substrates

Net absorption of DOACs is dependent on the intestinal permeability glycoprotein (P-gp) efflux transporter. Strong P-gp inducers, such as rifampin, older antiepileptics (carbamazepine, phenytoin, and phenobarbital), and St John’s wort, decrease exposure to DOACs and concurrent use should be avoided due to increased risk of thrombosis. Strong P-gp inhibitors such as amiodarone, verapamil, clarithromycin, dronedarone and antifungals (e.g. itraconazole, ketoconazole) increase the absorption, exposure and bioavailability of DOACs, potentially leading to increased bleeding complications. P-gp inhibitors increase dabigatran bioavailability by ~10% to 20% [71]. There are also case reports of major bleeding in elderly patients taking concomitant dabigatran with P-gp inhibitors that might have been due in part to the inhibition of P-gp, in addition to other factors such as age and decreased renal function [72, 73] (Table 1). More recently, in a retrospective cohort study of AF patients on dabigatran, concomitant use of digoxin, which is a substrate of P-gp, was associated with 33% increased risk of gastrointestinal bleeding (Table 1) [74].

##### CYP450 inducers and inhibitors

Dabigatran is not a substrate, inhibitor, or inducer of hepatic CYPs [75]. As less than 4% of the active metabolite of edoxaban is metabolised by CYP3A4 [76], drug interactions with CYP inducers or inhibitors are not expected. However, rivaroxaban and apixaban are CYP3A4 substrates; co-administration with drugs that inhibit or induce this metabolic enzyme as well as P-gp (e.g. ketoconazole or rifampicin) could significantly affect drug response, leading to increased risk of bleeding or reduced efficacy, respectively [77, 78].

##### Anticoagulants, antiplatelet agents and nonsteroidal anti-inflammatory agents

The DOAC product monographs contraindicate the concomitant use of other anticoagulants due to an increased bleeding risk [79–82]. Caution is also warranted if co-prescribing DOACs and other drugs that elevate bleeding risk: these include antiplatelets, nonsteroidal anti-inflammatory drugs (NSAIDs), cyclooxygenase-2 inhibitors, and systemic corticosteroids (Table 1). A post hoc analysis of the RE-LY trial showed that the rate of major bleeding was higher in patients on concomitant antiplatelet drugs compared to those not on antiplatelet therapy [83] (Table 1). Furthermore, in patients on a DOAC and antiplatelet therapy, dual antiplatelet treatment was associated with a higher risk of major bleeding than single antiplatelet therapy [83] (Table 1). Subgroup analysis of the EINSTEIN-DVT and EINSTEIN-PE trials demonstrated that rivaroxaban-treated patients co-administered a NSAID had a 2.4-fold higher risk of a major bleed and those who concomitantly took aspirin had a 1.5-fold higher risk [84] (Table 1). Aspirin and NSAID use increased the risk of major bleeding in apixaban-treated patients by approximately 30% [85]. Patients with AF receiving antiplatelet therapy in addition to edoxaban had higher rates of bleeding and cardiovascular death than those not on antiplatelet therapy [86]. It is therefore important to consider the patient–specific risk-benefit profile when DOACs are prescribed with permissible agents that may increase bleeding risk, and concurrent therapy should be administered for the shortest appropriate duration.

### Weight

Although population pharmacokinetic-pharmacodynamic studies have shown that extremes of bodyweight (<50 or >110 kg) do not significantly impact dabigatran pharmacology [75], a post hoc analysis of the RE-LY trial [87] showed a 20% decrease in dabigatran trough levels in patients >100 kg compared to patients 50–100 kg; no drug label dose adjustments have been recommended though [88]. Increased BMI is strongly associated with increased glomerular filtration rates (GFRs) [89] and increased drug clearance [90]. Hence the inverse correlation between weight and dabigatran levels could impact efficacy in very obese patients [91]. Two case studies of incident ischaemic stroke [92, 93] have been reported in obese patients (BMI > 39 kg/m^2^) on long-term anticoagulation with dabigatran for AF. The two patients had sub-therapeutic trough dabigatran levels and supra-physiologic creatinine clearance [92, 93], suggesting fixed-dose dabigatran may be insufficiently effective in severe obesity.

A pharmacokinetic and pharmacodynamic assessment indicated that extremes of bodyweight have limited effect on the pharmacokinetic profile of rivaroxaban. This is most likely due to rivaroxaban’s low volume of distribution [94] (Table 2). Pre-specified subgroup analyses stratified by weight and/or BMI within large phase III rivaroxaban trials have shown that efficacy and safety outcomes are consistent between different-weighted rivaroxaban users [95–102], suggesting that fixed-dose rivaroxaban regimens can be used safely in patients of all weight ranges [103]. Case studies suggest that the bioavailability of rivaroxaban is not affected in patients who are obese or morbidly obese [93, 104] and dose adjustments seem unnecessary. Interestingly, in a clinical case report of an obese patient who presented with an ischaemic stroke whilst on dabigatran, substitution to rivaroxaban lead to peak and trough rivaroxaban levels consistent with effective anticoagulation [93], suggesting that rivaroxaban is more efficacious than dabigatran in obese patients with AF.

Extremes of body weight lead to modest changes in apixaban exposure [105], and so weight ≤60 kg is recommended as one of the two criteria for reduced apixaban dosing in the current apixaban label [80, 106]. Similarly, in phase II edoxaban studies of patients with AF, weight ≤60 kg was associated with increased edoxaban exposure [107] and possible increased bleeding incidence [108], leading to a dose reduction recommendation (30 mg once daily if ≤60 kg) [81, 109].

### Hepatic impairment

Patients can be classified into three distinct groups of liver diseases: Child-Pugh A (mild), B (moderate) and C (severe) based on the presence of encephalopathy or ascites, along with the levels of serum albumin, serum bilirubin, and prothrombin time. Patients with severe liver disease were excluded from the DOAC clinical trials as hepatic impairment is often associated with intrinsic coagulation abnormalities, leading to an increased bleeding risk.

Given that rivaroxaban, apixaban and edoxaban are metabolised by liver enzymes, hepatic impairment can considerably affect the disposition of these anticoagulants [110]. Moderately impaired liver function is associated with 2.27-fold increase in rivaroxaban exposure, which is paralleled by an increase in factor Xa inhibition [111]. Conversely, apixaban pharmacokinetics are not significantly altered in patients with mild to moderate hepatic impairment or in patients with alanine aminotransferase and aspartate aminotransferase levels >2× upper limit of normal (ULN) [112]. Peak serum edoxaban concentrations decreased by 10% and 32% in patients with mild and moderate hepatic impairment, respectively [113]. Product labelling for the three factor Xa inhibitors does not recommend their use in patients with moderate or severe hepatic impairment.

The pharmacokinetic profile of dabigatran is not affected in individuals with moderate hepatic impairment [114] but as subjects with severe liver disease were excluded from clinical trials of dabigatran, dabigatran is not recommended in patients with elevated liver enzymes (>2× ULN).

### Renal impairment

Approximately 77% of dabigatran, 36% of rivaroxaban, 27% of apixaban, and 50% of edoxaban are excreted by the kidneys as active drug [76, 115–117]. Expectedly, DOAC pharmacokinetic studies have demonstrated that renal impairment is associated with elevated systemic exposure. In patients with severe renal impairment, as defined by creatinine clearance (CrCl) < 30 mL/min, the plasma concentration area under the curve (AUC) of dabigatran, rivaroxaban, apixaban, and edoxaban were increased by 6-fold, 65%, 44% and 72%, respectively [118–121]. Product labelling of DOACs recommends dose reduction for patients with CrCl 15–50 mL/min and avoidance of use in patients with advanced renal dysfunction (CrCl <15 mL/min) and in those on haemodialysis [79–82]. A sub-group analysis of the ENGAGE AF-TIMI 48 clinical study suggested that edoxaban-treated patients with CrCL >95 mL/min are potentially at a slightly higher risk of stroke/systemic embolism compared to those treated with warfarin, but with lower bleeding risk [122]. As such, the edoxaban drug label recommends edoxaban is used only after a careful individualised assessment of thromboembolic and bleeding risks in patients with high CrCl.

### Elderly

Age and renal function are intricately related. GFR gradually declines with ageing at a rate of ~1 ml/min/year after the age of 30 [123], with an accelerated decrease in GFR after 65 years of age [124]. To ensure the efficacy and safety of DOACs in elderly patients, renal function should be monitored annually in those with CrCl >50 mL/min and 2 to 3 times per year in those with CrCl 30–49 mL/min [125].

Non-adherence is also of concern as DOACs have short half-lives and missed doses could decrease efficacy, increasing the risk of thromboembolic events. Other factors such as poly-pharmacy, cognitive impairment, hospitalisation, history of bleeding and/or falls are common in the elderly, which could lead to over- or under-dosing of DOACs.

### Genetic factors

Given that there are strong genetic effects associated with warfarin dosing requirements, there has also been interest in whether genetic factors may determine outcomes with the DOACs, A GWAS conducted in a subset of patients from the RE-LY trial reported genome-wide SNP associations for both peak and trough dabigatran concentrations [126]. The minor allele of rs8192935, an intronic SNP located in the carboxylesterase 1 gene (*CES1*), was associated with a 12% reduction in peak dabigatran concentrations. By contrast, the minor allele of rs4148738, an intronic SNP located in *ABCB1*, which encodes P-gp, was associated with a 12% increase in peak dabigatran concentrations (Table 1). However, neither rs8192935 nor rs4148738 were associated with clinical outcomes. Importantly, *CES1* rs2244613 was associated with both decreased dabigatran trough levels and a 33% lower risk of bleeding events per minor allele [126] (Table 1). These findings need replication but at present do not seem to of clinical value.

Genetic studies focusing on clinical outcomes with dabigatran and the anti-Xa inhibitors have usually been small scale with inconsistent findings [127, 128]. More recently, a larger study of 2364 patients treated with either apixaban and/or rivaroxaban, of whom 412 had clinically relevant non-major bleeding or major bleeding, evaluated eight functional variants in five genes (ABCB1, ABCG2, CYP2J2, CYP3A4, CYP3A5), and found that none of the genetic variants were associated with bleeding [129]. Older patients, those who switched from one DOAC to another, and those on P450 or Pgp inhibitors were at increased risk of bleeding.

From the limited number of studies conducted on genetic factors associated with DOAC-related clinical end-points, it can be concluded that no common variants with a large effect size have been identified. This contrasts with the findings with warfarin. It is possible that rare variants may be important and/or multiple common variants with a small effect size may determine outcomes, but these hypotheses will need to be tested in large well designed studies which focus on patients with major bleeding episodes, who will need to be sequenced (for rare variants) and assessed for polygenic scores.

Unpredictable, or type B, ADRs have been reported with DOACs, but these have been sporadic and no genetic studies have been undertaken. An older direct thrombin inhibitor ximelagatran was withdrawn because of liver injury. A genetic study involving 74 cases and 130 treated controls showed a strong association with the HLA alleles DRB1*07:01 and DQA1*02, suggesting an immune pathogenesis [130]. Liver injury was not found to be associated with the newer DOACs in a systematic review of 29 randomised trials evaluating over 150,000 patients [131]. However, a systematic review of 15 studies of patients who developed liver injury while taking DOACs suggested that hepatotoxicity can occur rarely, but the outcome is usually favourable [132].

### Other factors

Little is known about the risk factors associated with the DOAC efficacy and safety in real-world practice. Other than co-medications, genetics, weight, age, renal and hepatic function, factors such as gender, concomitant diseases, infections, and lifestyle variables (e.g. smoking, alcohol intake) may also play a role in the efficacy and safety of DOACs. Recent real-world data from a retrospective study investigating AF patients initiated on dabigatran found an increased risk of gastrointestinal bleeding in patients who were female, had congestive heart failure, had previous *H. pylori* infection, and were diagnosed with alcohol abuse [74]. In addition, a 67% increased risk of gastrointestinal bleeding was observed among patients with chronic kidney disease [74]

To date, the effect of ethnicity on the efficacy and safety of DOACs remains uncertain due to poor enrolment of black and Hispanic patients and inconsistent race/ethnicity reports in major DOACs clinical trials [133].

### Monitoring and antidotes

Although anticoagulation monitoring for DOACs is not mandated, assessment of drug exposure and anticoagulant effect may be beneficial in specific clinical situations such as those with renal or hepatic insufficiency, identifying potential drug-drug interactions, in cases of suspected overdosing, and in the presence of serious bleeding or thrombotic events. Given the unique mechanisms of action of DOACs, routine INR testing is unsuitable [134–137]. Suitable dose monitoring tests have been outlined in Table 2. Briefly, the diluted thrombin time (dTT) and ecarin clotting time (ECT) assays are sensitive to the magnitude of dabigatran’s anticoagulant effect and have a linear response to plasma dabigatran within its therapeutic range [138]. The activated partial thromboplastin time (aPTT) only gives an approximate assessment of dabigatran’s effect on coagulation [134–136, 139]. The chromogenic anti-Factor-Xa assay, calibrated to rivaroxaban, apixaban or edoxaban may be used to quantitatively assess for clinically relevant drug levels, but these assays are not as yet available worldwide [140–142].

Antidotes are now available for the reversal of DOACs in case of emergencies (Table 2). However, challenges in their usage in clinical practice are anticipated. Clear guidelines on timing of usage, indications, and bleeding types will be required for practitioners [143].

## Parenteral anticoagulants

### Unfractionated heparin

Heparin is an endogenously produced highly sulphated linear glycosaminoglycan (mucopolysaccharide). Clinically used UFH is derived from porcine or bovine mucosa, and is heterogeneous with respect to molecular size, pharmacokinetics and anticoagulant activity [144]. The mean molecular weight of UFH molecules is 15,000 Da (Table 3), corresponding to approximately 45 saccharide units, although UFH molecules range from 3000 to 30,000 Da [144]. A unique pentasaccharide sequence enables high-affinity binding of antithrombin (AT) to heparin molecules, converting AT from a slow into a rapid serine protease inhibitor [145]. However, this pentasaccharide sequence is only present in a third of UFH molecules, and heparin molecules lacking this sequence have reduced anticoagulant activity at therapeutic levels [144, 146]. The heparin/AT complex inactivates the main recognised physiological targets of AT: thrombin (factor IIa) and factor Xa [147] (Fig. 2). Inhibition of thrombin requires formation of a ternary heparin/AT/thrombin complex; heparin chains less than approximately 18 saccharide units are not long enough to bridge AT to thrombin, and so have little anti-IIa activity [148]. However, anti-Xa activity only requires heparin to bind to AT, and so shorter heparin molecules that contain the pentasaccharide sequence still catalyse factor Xa inhibition [144]. UFH also has additional mechanisms of action, detected in in vitro studies, including AT-dependent inhibition of factors IXa, XIa and XIIa, and at high concentrations pentasaccharide sequence-independent heparin cofactor II (HCII)-dependent inhibition of factor IIa [144].

**Table 3:** Pharmacological characteristics of unfractionated and low molecular weight heparins.

Characteristic	Unfractionated heparin (UFH)	Tinzaparin	Dalteparin	Enoxaparin	Nadroparin
Trade name	Heparin	Innohep	Fragmin	Clexane	Fraxiparine
Mean molecular mass (kDa)	15	6.8	6.0	4.5	4.3
Dose-dependent pharmacokinetics	Yes	No	No	No	No
Administration route	Intravenous & subcutaneous	Subcutaneous	Subcutaneous	Intravenous & subcutaneous	Intravenous & subcutaneous
Bioavailability after SC injection (%)	Low doses: 10 High doses: 90	86.7	87	92	89
Protein/cell binding	Binds to macrophages, endothelial cells, platelets and many plasma proteins (e.g. lipoproteins, von Willebrand factor)	Reduced, but still occurs	Reduced, but still occurs	Reduced, but still occurs	Reduced, but still occurs
Mean peak activity after SC injection	2–4	4–6	4	3–5	3–6
Volume of distribution (L)	4	4	3	5	3.6
Elimination *t*_1/2_ after SC injection	1–2.5	3–4	3–4	4	3.5
Main elimination route	Low doses: cellular (liver) High doses: renal	Renal	Renal	Renal	Renal
Monitoring	aPTT	Not routinely indicated (anti-Xa activity)	Not routinely indicated (anti-Xa activity)	Not routinely indicated (anti-Xa activity)	Not routinely indicated (anti-Xa activity)
Pharmacodynamic anti-Xa/IIa ratio	1	2.8	2.2	>4.0	3.5
Intra- and interindividual variability	Extensive	Reduced, but still present	Reduced, but still present	Reduced, but still present	Reduced, but still present
References	[144, 149, 270–272]	[218, 272]	[217, 272]	[220, 272]	[219, 272]

*aPTT* activated partial thromboplastin time, *SC* subcutaneous.

At therapeutic doses, UFH clearance is nonlinear with dose-dependent pharmacokinetics involving both saturable and non-saturable elimination mechanisms [149]. The rapid saturable component is the main clearance route of UFH and involves cellular uptake and metabolism by liver sinusoidal endothelial cells, which constitute part of the hepatic reticuloendothelial system (RES), and/or by vascular endothelial cells; the slower non-saturable component is largely renal [149]. UFH also binds to endothelial cells, macrophages, platelets and multiple plasma proteins besides AT including von Willebrand factor, lipoproteins and fibrinogen, which limits the anticoagulant potency of UFH and increases the variability in response to UFH [144, 150]. Although use of UFH has declined, it is still used in patients with acute coronary syndrome undergoing percutaneous coronary intervention (PCI) [151], and in clinical settings where anticoagulation fine tuning is sought (e.g. in perioperative anticoagulant bridging or in patients at high bleeding risk) because of its rapid onset of action and clearance, and the availability of protamine sulphate for rapid UFH inactivation [152].

#### Factors affecting heparin efficacy

As anticoagulant response to UFH is unpredictable, UFH therapy is monitored mainly using the aPTT, although the activated clotting time (ACT) is used to monitor the higher UFH doses administered in PCI and cardiopulmonary bypass surgery [144]. The evidence behind the aPTT therapeutic range used is relatively weak (1.5–2.5× the control level or upper limit of normal [32–39 s] [152]), as it has not been verified in randomised trials [144], and patients with a prolonged baseline aPTT cannot have UFH therapy reliably monitored using aPTT [152]. Clinically, failure for rapid attainment of a therapeutic aPTT after starting UFH has been associated with VTE recurrence in some [153], but not all studies [154]. Interestingly, the risk of 180-day VTE recurrence following an incident VTE was reduced in patients who rapidly attained an aPTT ≥ 58 s on UFH, but not in patients with rapid attainment of aPTT ≥40 s [155]. Conversely, during the median six day duration of UFH therapy, the proportion of time with an aPTT ≥40 s, but not ≥58 s, was associated with a reduced hazard of VTE recurrence [155]. Markedly low aPTTs (<1.25x control) taken 4–6 h after starting UFH therapy have also been associated with recurrent myocardial infarction [156].

A barrier to the rapid attainment of a therapeutic aPTT is under-dosing of both UFH loading and infusion maintenance doses [157]. Thus UFH nomograms have been developed, which significantly increase the proportion of patients reaching a therapeutic aPTT within 24 h compared to clinical judgement [158]. UFH nomograms standardise the loading and initial heparin infusion rate and provide an algorithm for rate adjustments based on aPTT measurements; both weight and non-weight based nomograms are available [152]. Nevertheless, in a RCT sub-analysis including 6,055 patients with a ST-elevation myocardial infarction who received UFH according to a weight-based nomogram, only 33.8% of initial aPTTs fell within the therapeutic range; 13.2% and 16.3% were markedly low and high, respectively [156]. Factors associated with markedly low initial aPTT values on UFH included increased weight and younger age [156]. Even when the initial aPTT on UFH using a nomogram is within the therapeutic range, it is maintained over the next two measurements in only 29% of patients [159].

### Heparin resistance

Heparin resistance refers to the requirement for unusually high heparin doses to achieve a therapeutic aPTT, and studies have suggested that it occurs in 21–26% of patients [160, 161]. Several factors have been associated with heparin resistance including nonspecific binding secondary to strong negative charge, AT deficiency, platelet count >300,000/microL, recent heparin therapy, increased levels of heparin-binding proteins, increased heparin clearance, high levels of factor VIII and fibrinogen and concomitant use of the serine protease inhibitor, aprotinin [144, 160, 162–164]. Given the importance of ascertaining an early therapeutic aPTT, further research is required to incorporate factors associated with a decreased response into UFH nomograms.

#### Factors associated with heparin safety

##### Bleeding

Major bleeding occurs in up to 7% of patients exposed to therapeutic UFH [165]. Risk factors for heparin-associated bleeding include older age, female gender, recent surgery or trauma, hepatic dysfunction, haemostatic problems, peptic ulcer disease, malignancy, reduced admission haemoglobin and concomitant use of other anti-clotting agents (e.g. antiplatelet drugs and thrombolytics) [152, 165–167]. Independent risk factors associated with markedly elevated initial aPTT values on UFH (≥2.75 times control) are older age, female sex, lower weight and renal dysfunction [156]. However, aPTT values correlate inconsistently with UFH-associated bleeding [156, 165, 167] and patients can suffer serious bleeding when the aPTT is in the therapeutic range, indicating that underlying clinical predictors appear stronger bleeding risk factors than aPTT [165]. No significant differences in bleeding rates have been observed in patients administered UFH according to nomograms, compared to non-nomogram dosing [158].

##### Heparin-induced thrombocytopaenia

HIT is an antibody-mediated ADR. Antibody formation, thrombocytopaenia and thrombosis occur in up to 8%, 1–5% and 0.2–1.3% of heparin-exposed patients, respectively [168]. Thrombosis in HIT is associated with a 20–30% risk of mortality [169]. The pathophysiology of HIT involves heparin binding to platelet factor 4, subsequent autoantibody production, and then the binding of IgG autoantibodies to the platelet surface stimulating platelet activation [170].

The risk of developing HIT is greater with UFH than LMWH for surgical patients [171], although this has not been confirmed in medical patients [172]. Therapeutic dose UFH poses an elevated risk of HIT compared to prophylactic dose UFH [173], and female patients are at higher risk of HIT [174]. The 4Ts pre-test clinical scoring system has been developed that incorporates thrombocytopaenia, the timing of platelet count fall, thrombosis, and other possible causes for observed thrombocytopaenia. Whilst the 4Ts score has an excellent negative predictive value, its positive predictive value remains suboptimal [175].

Genetic factors may be important in predisposing to HIT. Early candidate gene studies suggested some associations [176], which were not replicated, including an association of the homozygous 131RR genotype in the IgG receptor IIa gene, *FCGR2A*, with thrombosis in HIT patients [177]. More recently, genome-wide approaches have also been utilised. Karnes et al. in a study comparing 67 HIT cases with 884 heparin-exposed controls, reported that SNPs near the T-cell death-associated gene 8 (*TDAG8*) are associated with HIT in a recessive model, with the strongest association for the imputed SNP, rs10782473, with an OR 18.52 (95% CI 6.33–54.23) [178]. The most strongly associated genotyped SNP, rs1887289, leads to decreased *TDAG8* transcription in cis-expression quantitative trait loci (eQTL) studies of healthy individuals, and is in moderate linkage disequilibrium with a *TDAG8* missense SNP (rs3742704) [178].

More recently, a larger GWAS comparing anti-PF4 antibody positive patients who were also positive in the functional assay (*n* = 1269) with antibody positive functional assay-negative controls (*n* = 1131) and antibody negative controls (*n* = 1766) showed an association with the ABO blood group locus, with the O blood group being identified as a risk factor (OR, 1.42; 95% CI, 1.26–1.61; *P* = 3.09 × 10^−8^) for thrombosis in HIT [179]. Since the blood group is already known in most patients, there should perhaps be extra caution in blood group O patients who develop thrombocytopenia on heparin treatment. A subsequent GWAS that investigated the association with anti-PF4/heparin antibodies returned no genome-wide significant hits [180].

##### Hyperkalaemia

Heparin can lead to reversible hypoaldosteronism, resulting in a decrease in blood sodium and increase in potassium levels [181], which can predispose to hyperkalaemia. The most important mechanism appears to be a decrease in both the number and affinity of angiotensin II receptors in the zona glomerulosa [182]. Serum potassium levels above the upper limit of normal occur in ~7% of patients on heparin [182] and usually occur within 14 days of initiating heparin therapy [182]. The risk of hyperkalaemia appears higher with UFH than LMWHs [183]. Heparin-associated hyperkalaemia usually requires the presence of additional risk factors that perturb potassium homoeostasis including diabetes mellitus, metabolic acidosis, renal dysfunction and concomitant medications including spironolactone, angiotensin-converting enzyme inhibitors, trimethoprim and nonsteroidal anti-inflammatory drugs [182–185].

##### Low molecular weight heparins

LMWHs are derived from UFH through chemical or enzymatic depolymerisation, have approximately one third the molecular weight of UFH (Table 3), and have largely superseded UFH. LMWHs have reduced anti-IIa activity relative to anti-Xa activity because of their shorter molecular length (mean weight corresponds to ~15 saccharide units [144]), a greater bioavailability and a longer duration of anticoagulant effect than UFH, permitting once/twice daily dosing. Although molecular and thus pharmacological heterogeneity still exists within and between LWMHs, it is less pronounced than for UFH, meaning LMWHs are routinely prescribed without monitoring. In VTE treatment, LMWH is used for both rapid anticoagulation during warfarinisation (LMWH therapy continuing until a stable therapeutic INR has been achieved), and when heparin-based anticoagulation is indicated for the duration of VTE treatment (e.g. in pregnancy). The dosing of LMWHs is mostly fixed for VTE thromboprophylaxis, but is weight-based for VTE treatment.

#### LMWH anti-Xa monitoring

Although LMWH therapy is generally unmonitored, monitoring has been suggested in specific clinical settings including adult patients receiving LMWH with concomitant renal dysfunction [186, 187], morbid obesity, during pregnancy, and to check compliance [188, 189]. Consensus-based paediatric guidelines also recommend monitoring therapeutic LMWH in paediatric patients [190]. The recommended monitoring test is the chromogenic anti-Xa assay, which indirectly determines drug concentration (in anti-Xa International Units/mL) by measuring ex vivo the extent to which exogenous factor Xa is inhibited by LMWH-antithrombin complexes present in the patient’s blood sample. Clinical factors associated with anti-Xa activity on LMWH include dose, body weight [191, 192], renal function [193] (see later) and levels of tissue factor pathway inhibitor (TFPI) [194] and TFPI-Xa complexes [194]; the latter two being consistent with heparin-induced TFPI mobilisation [194].

The anti-Xa assay has limitations. Anti-Xa prophylactic and therapeutic index reference ranges are based on expert opinion rather than large prospective trial evidence [186, 187] and are different for different LWMHs, dosing schedules (once vs twice daily dosing) and indications (thromboprophylaxis vs treatment) [187]. Measured anti-Xa activity is affected by the timing of blood collection and interassay variation [195]. Thus, different assays can lead to different clinical decisions regarding optimal dosing in patients on the same LMWH [195]. Greater assay standardisation or assay-specific anti-Xa reference ranges are required.

Importantly, although anti-Xa levels are a marker of LMWH blood concentration, the correlation with clinical endpoints (bleeding, VTE) is inconsistent. For instance, elevated anti-Xa levels have been inconsistently correlated with bleeding [196–198], while a negative correlation was found between anti-Xa levels and VTE [198], but other studies found no association [197, 199]. Similarly, anti-Xa activity while on enoxaparin has been associated with increased risk of death or recurrent myocardial infarction, but not bleeding, in one study (*n* = 803) of acute coronary syndrome patients [200], whilst in a RCT sub-analysis of patients undergoing elective PCI (*n* = 2298), anti-Xa activity was associated with bleeding, but not death, myocardial infarction or revascularisation [201]. Besides variable definitions of supra- and subtherapeutic anti-Xa activity and the small sample sizes of many studies, the overall lack of reliable associations between anti-Xa activity and clinical events may plausibly be because the global anticoagulant effect of LMWHs involves additional factors besides anti-Xa activity, including anti-IIa activity, platelet levels, and interindividual variations in heparin-binding proteins [202–204].

#### LMWH VTE thromoboprophylaxis in critically ill trauma and surgical patients

Although anti-Xa monitoring has limitations, VTE thromboprophylaxis in patients at higher risk of VTE, principally critically ill trauma and surgical patients, *may* benefit from anti-Xa level monitoring, and in particular 12-h post dose/trough monitoring. These patients frequently have suboptimal anti-Xa trough levels [205, 206], and peripheral oedema is associated with reduced anti-Xa exposure [205]. Low body weight and multiple organ dysfunction have also been associated with high and low peak anti-Xa levels in intensive care patients, respectively [206]. A study of critically ill trauma and surgical patients reported that patients with low 12 h anti-Xa levels (≤0.1 IU/mL) on a VTE thromboprophylaxis regimen of enoxaparin 30 mg twice daily had an increased risk of DVT [207]. Interestingly in this study, peak anti-Xa levels were not different in those who did and did not develop a DVT [207]. A recent study of dalteparin VTE thromboprophylaxis in high risk trauma patients demonstrated that following transition to anti-Xa monitoring, VTE incidence decreased, and that in patients with 12-h anti-Xa levels available, those with levels <0.1 IU/mL had an increased risk of developing DVT [208]. However, this study also found that increased body weight partially correlated with low anti-Xa activity. Nevertheless, 12-h/trough anti-Xa monitoring of LMWH for VTE thromboprophylaxis in high risk critically ill patients merits further investigation.

#### Body weight

In general, patients at the extremes of body weight have been under-represented in LMWH RCTs. Although anti-Xa activity is inversely correlated to body weight [191, 192], weight accounts for only 16% of interindividual anti-Xa activity [196]. Nevertheless, 85% of patients receiving prophylactic enoxaparin who are under ≤45 kg of weight have anti-Xa activity ≥0.5 IU/mL [191], which is above the LMWH anti-Xa thromboprophylaxis accepted range for prophylaxis (0.2–0.5 IU/mL [209]). Nevertheless, the mean anti-Xa level was 0.64 IU/mL, which is still at the low end of the therapeutic anti-Xa range (0.5–1.2 IU/mL [209]) [191]. 54% of patients <50 kg have been reported to receive treatment LMWH therapy in excess of 200 IU/kg/day, compared to only 21% of patients weighing 50–100 kg [210]. Furthermore, weighing <50 kg was significantly associated with a higher rate of bleeding complications, although the extent to which this is attributable to low body weight per se remains unclear [211]. Larger studies are required to further investigate the interaction between treatment dose LMWH and low body weight on bleeding risk.

Excessive body weight is itself associated with an increased risk of primary and recurrent VTE [212]. Fixed doses in obese patients correlate with lower anti-Xa activity [192]. A weight based regimen for prophylactic enoxaparin dosing in medically hospitalised severely obese patients (BMI ≥ 40 kg/m^2^) in a small study (*n* = 31) significantly improved the proportion of patients with peak anti-Xa levels in the prophylactic therapeutic range [213]. A retrospective analysis of patients undergoing orthopaedic surgery (*n* = 817) on 40 mg daily fixed dose prophylactic enoxaparin reported that venographically detected VTE occurrence was significantly higher in obese compared to non-obese patients [214]. In sub-analyses of an RCT (*n* = 3706) comparing fixed dose prophylactic dalteparin to placebo in medical patients, a trend for benefit with dalteparin was present for all BMI categories except for patients with a BMI ≥ 40 kg/m^2^, suggesting that fixed dose LMWH thromboprophylaxis may be insufficient in severely obese patients [215]. However, within this study, the overall frequency of thrombotic and haemorrhagic events did not differ between obese (defined as BMI ≥ 30 kg/m^2^ for men and ≥28.6 kg/m^2^ for women) and non-obese patients [215]. For patients on treatment dose of either enoxaparin or UFH for VTE (*n* = 2217) in another RCT, patients weighing >100 kg (compared to patients ≤100 kg), and patients whose BMI was ≥30 kg/m^2^ (compared to those with BMI < 30 kg/m^2^) did not have a significantly increased risk of VTE recurrence or major bleeding [216].

The product monographs recommend capping maximum daily doses to 18,000 IU (dalteparin) [217], 28,000 IU (tinzaparin) [218], 19,000 IU (nadroparin) [219] and to 18,000 IU and 20,000 IU for once and twice daily dosing of enoxaparin, respectively [220]. There is still limited clinical data available to determine whether dose capping in clinical practice reduces therapeutic LMWH efficacy. Alternatively, there has been concern that increasing LMWH doses based on total body weight in obese patients may predispose to higher-than-predicted anti-Xa levels, with a potential bleeding risk. This is because LMWHs accumulate predominantly in the blood and vascular tissue, and intravascular volume is not linearly related with total body weight [221]. Nevertheless, a recent systematic review summarised the results of four pharmacokinetic studies of LMWH in obesity and concluded that dosing by total body weight does not lead to elevated anti-Xa levels in obese patients; the maximum body weight of a participant was 192kg [222]. Overall, studies involving larger numbers of severely obese patients are required to improving LMWH dosing in this group who are at high risk of thrombotic events.

#### Renal dysfunction

Renal elimination is preferentially more important to LMWHs than UFH, although the extent of its role in LMWH clearance, compared to cellular metabolism, varies between LMWHs. LMWHs of lower molecular weight (e.g. nadroparin, enoxaparin) preferentially rely on renal elimination whereas higher molecular weight LMWHs (e.g. tinzaparin) concomitantly utilise the cellular route of elimination. Interestingly, the affinity of LMWH fragments for antithrombin also influences elimination pathway propensity, with higher affinity fragments being preferentially eliminated by the cellular saturable route [149].

The major LMWH RCTs generally excluded patients with renal dysfunction. However, clinical studies have reported that enoxaparin anti-Xa exposure in non-haemodialysis patients with CrCl ≤ 30 mL/min is increased at both prophylactic and therapeutic doses [193]. Therapeutic nadroparin accumulates with decreasing renal function [223], but no accumulation was observed with prophylactic nadroparin in patients with a GFR of 30–50 mL/min [224]. No anti-Xa activity accumulation has been determined at prophylactic [193, 225] or therapeutic doses [226, 227] for dalteparin or tinzaparin in renal dysfunction. Interestingly in patients on haemodialysis, no anti-Xa accumulation with prophylactic enoxaparin or prophylactic dalteparin [228] was observed, suggesting that renal replacement therapy removes enoxaparin/dalteparin [193].

Although prophylactic enoxaparin is weakly associated with higher anti-Xa levels in patients with renal dysfunction [229], no excess bleeding has been confirmed, and anti-Xa levels have not differentiated between those with and without serious bleeding events [229]. Importantly, a meta-analysis of 12 studies (*n* = 4971) found that therapeutic enoxaparin is associated with an increased risk of major bleeding in patients with CrCl ≤ 30 mL/min compared to those with CrCl > 30 mL/min [230]. However, empirical dose reduction of therapeutic enoxaparin in patients with CrCl ≤ 30 mL/min may negate this elevated bleeding risk [230]. Therefore, in patients with renal dysfunction requiring therapeutic LMWH, a reduced enoxaparin dose [187], dalteparin, tinzaparin or UFH appear reasonable selections.

## Conclusions

The goal of anticoagulation therapy, whether oral or parenteral, is to safely shift the coagulation system equilibrium further from thrombogenesis in patients with either a regional hypercoagulable (e.g. AF, mechanical heart valve) or systemic hypercoagulable (e.g. antiphospholipid syndrome) predisposition. Although effective, anticoagulation therapy is associated with both thrombotic and haemorrhagic ADRs, as well as unpredictable ADRs. Currently, 50–60% of observed INR variability can be explained in patients on warfarin, with the majority attributable to genetic variation in *VKORC1* and *CYP2C9*. However, whilst the relationship between INR and clinical events is well characterised with respect to the use of warfarin, associations between aPTT or anti-Xa levels and clinical outcomes in patients on UFH or LMWHs, respectively, appear less clear and need further investigation. Furthermore, although factors have been associated with interindividual variation in response to DOACs (e.g. weight, and renal function), UFH (e.g. weight) and LWMHs (e.g. weight, and renal function for enoxaparin), the majority of observed variation in monitoring assays and clinical outcomes remains unexplained. Therefore, further research and large-scale anticoagulation therapy studies are required, especially considering their widespread and increasing use and the potential severity of adverse effects (bleeding or thrombosis). Priority research areas include: determining if extreme DOAC systemic exposures are associated with adverse clinical outcomes, conducting larger studies involving patients typically excluded from anticoagulation RCTs (e.g. at the extremes of weight, renal dysfunction), identifying novel biomarkers associated with differential anticoagulant response via systematic utilisation of omics- technologies (e.g. genomics, proteomics, metabolomics), and development of better methods to improve warfarin anticoagulation in under-served populations where usage is high. Large scale studies powered for clinical endpoints would be ideal and would help resolve the uncertainties arising from conflicting smaller studies. However, well designed studies using established anticoagulation biomarkers such as INR and anti-Xa would also be acceptable, and are likely to be cheaper and smaller than clinical end-point studies. Larger trials will most likely need international collaboration which inevitably will increase cost and complexity. Ultimately, clinicians strive for *primum non nocere* (‘first, do not harm’). This is highly relevant with anticoagulation where therapy is aiming to strike a fine balance between bleeding and thrombotic risks. Precision anticoagulant prescribing through better choice of either dose and/or drug may help in achieving this balance, but unfortunately, we are ‘not there yet’.

## Data Availability

This is a review article and all the relevant papers used for the review have been cited. No additional original data was used in the writing of the review, and therefore no specific data needs to be made available.
